# A machine learning model for predicting deterioration of COVID-19 inpatients

**DOI:** 10.1038/s41598-022-05822-7

**Published:** 2022-02-16

**Authors:** Omer Noy, Dan Coster, Maya Metzger, Itai Atar, Shani Shenhar-Tsarfaty, Shlomo Berliner, Galia Rahav, Ori Rogowski, Ron Shamir

**Affiliations:** 1 Blavatnik School of Computer Science, Tel-Aviv University, 30 Haim Levanon Street, 69978 Tel Aviv, Israel; 2 Sackler Faculty of Medicine, Tel-Aviv University, Tel Aviv, Israel; 3 Departments of Internal Medicine “C”, “E”, Tel-Aviv Sourasky Medical Center, Tel Aviv, Israel; 4 Infectious Diseases Unit, Sheba Medical Center, Ramat Gan, Israel

**Keywords:** Prognosis, Infectious diseases, Machine learning, Predictive markers

## Abstract

The COVID-19 pandemic has been spreading worldwide since December 2019, presenting an urgent threat to global health. Due to the limited understanding of disease progression and of the risk factors for the disease, it is a clinical challenge to predict which hospitalized patients will deteriorate. Moreover, several studies suggested that taking early measures for treating patients at risk of deterioration could prevent or lessen condition worsening and the need for mechanical ventilation. We developed a predictive model for early identification of patients at risk for clinical deterioration by retrospective analysis of electronic health records of COVID-19 inpatients at the two largest medical centers in Israel. Our model employs machine learning methods and uses routine clinical features such as vital signs, lab measurements, demographics, and background disease. Deterioration was defined as a high NEWS2 score adjusted to COVID-19. In the prediction of deterioration within the next 7–30 h, the model achieved an area under the ROC curve of 0.84 and an area under the precision-recall curve of 0.74. In external validation on data from a different hospital, it achieved values of 0.76 and 0.7, respectively.

## Introduction

The coronavirus disease 2019 (COVID-19) emerged in China in December 2019, and since then has spread rapidly around the world. In March 2020, the World Health Organization declared the COVID-19 outbreak as a global pandemic^1^. As of June 2021, worldwide cases exceeded 172 million and more than 3.5 million died^2^. The extent of the disease varies from asymptomatic to severe, characterized by respiratory and/or multi-organ failure and death^3,4^. Healthcare systems worldwide have faced an overwhelming burden of patients with COVID-19. At the same time, there is limited understanding of disease progression, risk factors for deterioration, and the long-term outcomes for those who deteriorate. Moreover, early treatments such as antiviral medications may prevent clinical deterioration in COVID-19 patients^5^. Therefore, early warning tools for COVID-19 deterioration are required. Tools that predict deterioration risk in individuals can also improve resource utilization in the clinical facility and its wards, by aggregating risk scores of patients for anticipating expected changes in patient load^6^.

Prognostic scores for clinical deterioration of patients are widely used in medicine, particularly in critical care. The National Early Warning Score 2 (NEWS2), the quick Sequential Organ Function Assessment (qSOFA), and CURB-65^7–9^ are commonly used clinical risk scores for early recognition of patients with severe infection. The NEWS2 score incorporates pulse rate, respiratory rate, blood pressure, temperature, oxygen saturation, supplemental oxygen, and level of consciousness or new confusion. Liao et al.^10^ suggested an early warning score for COVID-19 patients termed “modified-NEWS2” (mNEWS2). It adds to the NEWS2 formula the factor age $\ge$ 65 years, reflecting the observation that increased age is associated with elevated risk for severe illness (Supplementary Table 1).

Machine learning methods integrate statistical and mathematical algorithms that enable the analysis of complex signals in big-data environments^11,12^. In recent years, such methods were shown to be highly effective for data-driven predictions in a multitude of fields, including healthcare^12^. They enable rapid analysis of large electronic health records (EHRs) and can generate tailored predictions for each patient. As a consequence, machine learning methods have great potential to help improve COVID-19 care.

We developed a machine learning model for early prediction of deterioration of COVID-19 inpatients, defined as mNEWS2 score $\ge$ 7. The model was developed by analyzing longitudinal EHRs of COVID-19 inpatients in Sheba Medical Center (Sheba), the largest hospital in Israel. To validate the generalizability of its performance, we applied our model on EHRs of inpatients diagnosed with COVID-19 from the second largest hospital in Israel, the Tel-Aviv Sourasky Medical Center (TASMC).

## Results

### Cohort description

We conducted a retrospective study on two cohorts. The *development cohort* consisted of EHRs of all COVID-19 positive adults admitted to Sheba between March and December 2020. The *validation cohort* consisted of EHRs of all COVID-19 positive patients admitted to TASMC between March and September 2020. The data used was extracted from structured longitudinal EHRs covering the entire hospitalization period, starting from the hospital admission. The data included both time-independent (static) and temporal (dynamic) features, such as demographics, background disease, vital signs and lab measurements (Supplementary Table 2). We use the term *observation* for the vector of hourly aggregated feature values of a patient. A new observation was formed whenever at least one measurement was recorded in that hour.

After applying the inclusion and exclusion criteria (see "Methods"), the development set contained 25,105 hourly observations derived from 662 patients; the validation set had 7,737 observations derived from 417 patients. The characteristics of the first measurements upon admission of the datasets are described in Supplementary Table 2.

We defined the deterioration outcome as a recorded high mNEWS2 score (≥ 7), and aimed to predict such outcomes 7–30 h in advance (Supplementary Fig. 1). Higher mNEWS2 scores were associated with higher mortality and ICU admissions rates in the development dataset (Supplementary Fig. 2).

### COVID-19 deterioration model

Our models predict the risk of deterioration for each hour that contains a new observation. The development set was split into *training* and *testing sets* (Supplementary Fig. 3), where the training set consisted of 20,029 hourly observations derived from 530 patients, of which 6,349 (~ 31%) were labeled positive (mNEWS2 $\ge$ 7 in the next 7–30 h). We trained 14 models on the training set.

**Figure 1:**
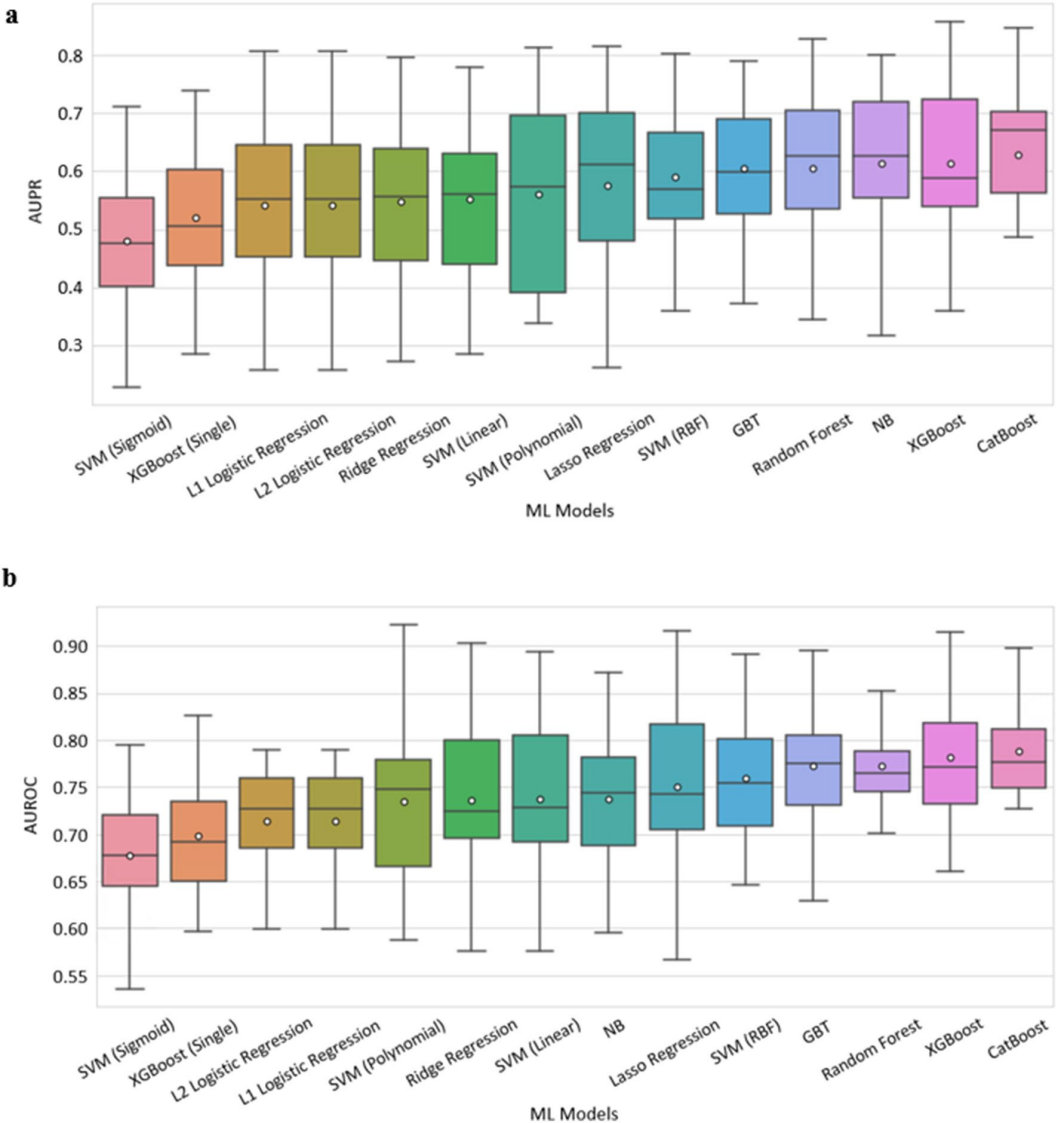
Performance of 14 machine learning models that predict mNEWS2 ≥ 7. Comparison of machine learning methods using 20-fold cross-validation over the training set within the development dataset. (**a**) AUPR. (**b**) AUROC. The horizontal line indicates the median, and the white circle indicates the mean. The models are sorted by the mean AUC.

**Figure 2:**
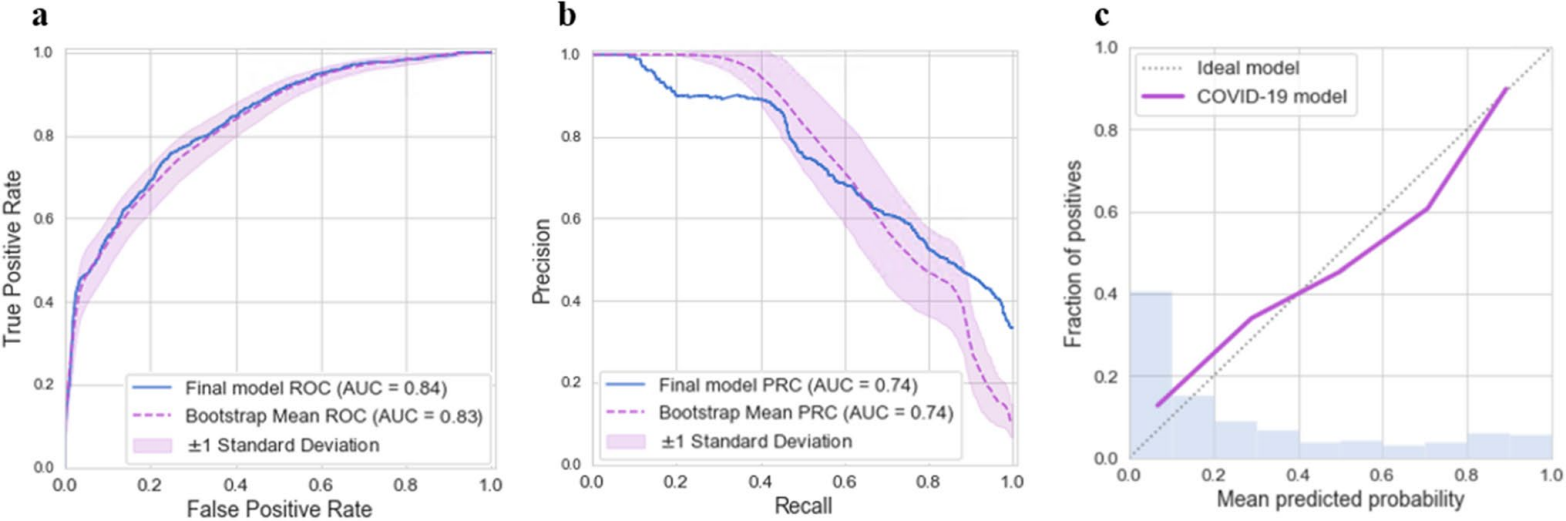
Performance of the final model on the testing set within the development set. (**a**) AUROC. (**b**) AUPR. Solid curves were computed on the total set. Dashed curves were computed with a bootstrap procedure with 100 iterations, where, in each iteration, 50% of the testing set was sampled with replacement. (**c**) Calibration plot for the relationship between the predicted and observed probabilities for COVID-19 deterioration. The dashed diagonal line represents an ideal calibration. The purple line represents the actual model performance in five discretized bins. The blue histogram is the distribution of the risk predictions.

Figure 1 summarizes the performance of 14 classifiers in cross-validation on the training set. All predictions refer to events at least seven hours in advance. Classifiers based on an ensemble of decision trees (CatBoost, XGBoost, Random Forest) performed best overall. We chose CatBoost as our final prediction model and trained it on the entire training set. Its results on the development testing set are shown in Fig. 2. It had good discrimination and achieved AUROC of 0.84 and AUPR of 0.74. To estimate the robustness of the model, we performed a bootstrap procedure with 100 iterations, where, in each iteration, a sample containing 50% of the testing set was randomly selected with replacement. The mean and standard deviation of the AUROC and the AUPR over these experiments achieved comparable results to those of the total testing set (Fig. 2a-b). Figure 2c presents a calibration curve of the model, showing good agreement between the predicted and observed probabilities for deterioration.

When using a classification threshold of 0.7 in the final model (namely, classifying as positive all observations with risk score $>$ 0.7, and the rest as negative), it achieved an accuracy of 80% with a positive predictive value (PPV) of 87% on the testing set. Performance metrics for various classification thresholds are shown in Supplementary Table 3.

**Figure 3:**
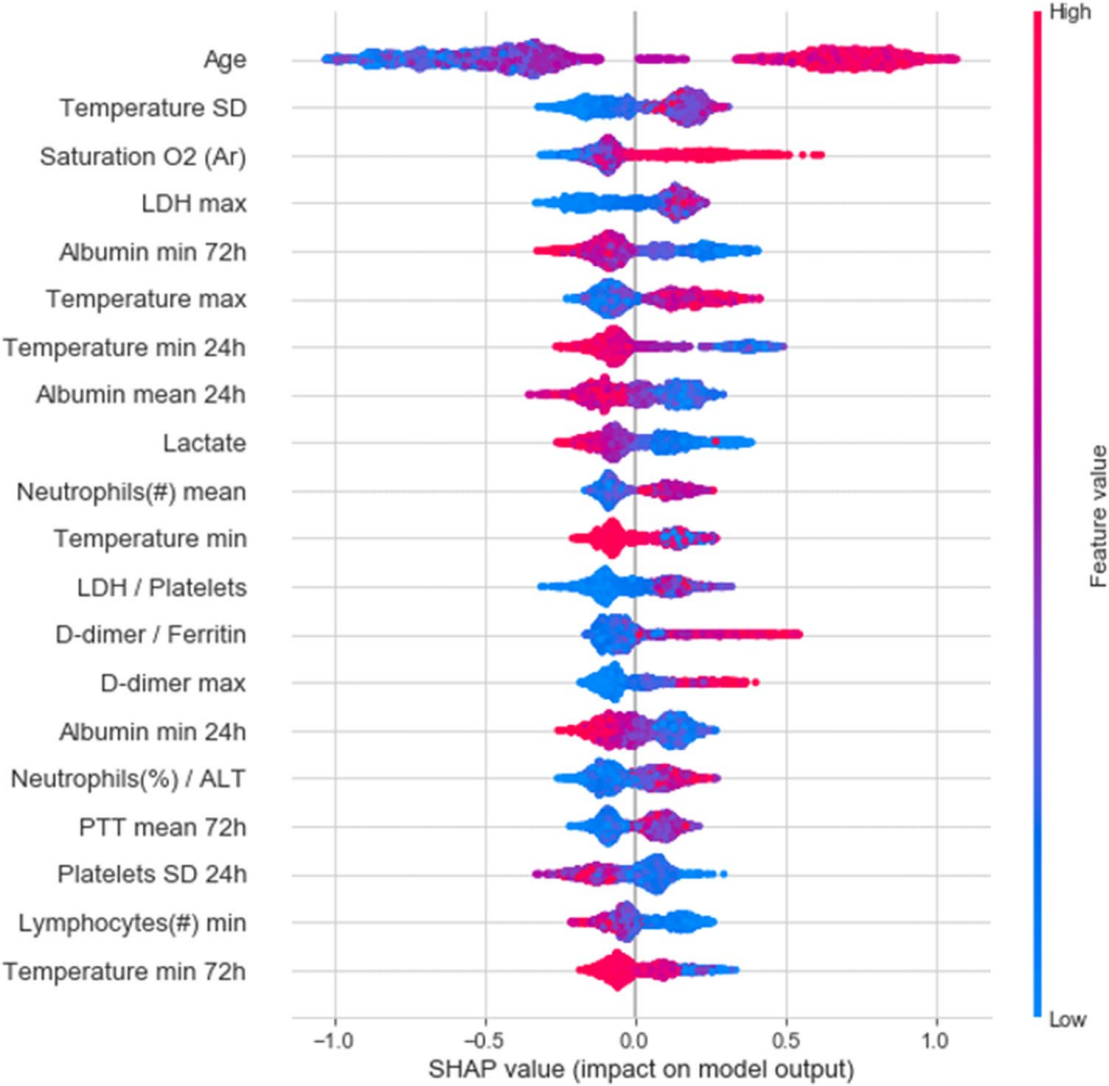
20 features with highest mean absolute SHAP values. Features (rows) are ordered in decreasing overall importance to the prediction. The plot for each feature shows the SHAP value for each observation on the x-axis, with color representing the value of the feature from low (blue) to high (red). The absolute value indicates the extent of the contribution of the feature, while its sign indicates whether the contribution is positive or negative. SD: standard deviation; /: the ratio between two features. 24 h,72 h: time windows within the statistic was computed. If not mentioned, the statistics is calculated on the entire hospitalization period so far.

To assess the contribution of each feature to the final model prediction, we used SHAP values^13^. The top 20 important features of the model are summarized in Fig. 3. Age, arterial oxygen saturation, maximal LDH value and the standard deviation of body temperature were the most important features for predicting deterioration. An evaluation of feature importance as calculated by the CatBoost algorithm gave similar results (Supplementary Fig. 4).

### External validation

**Figure 4:**
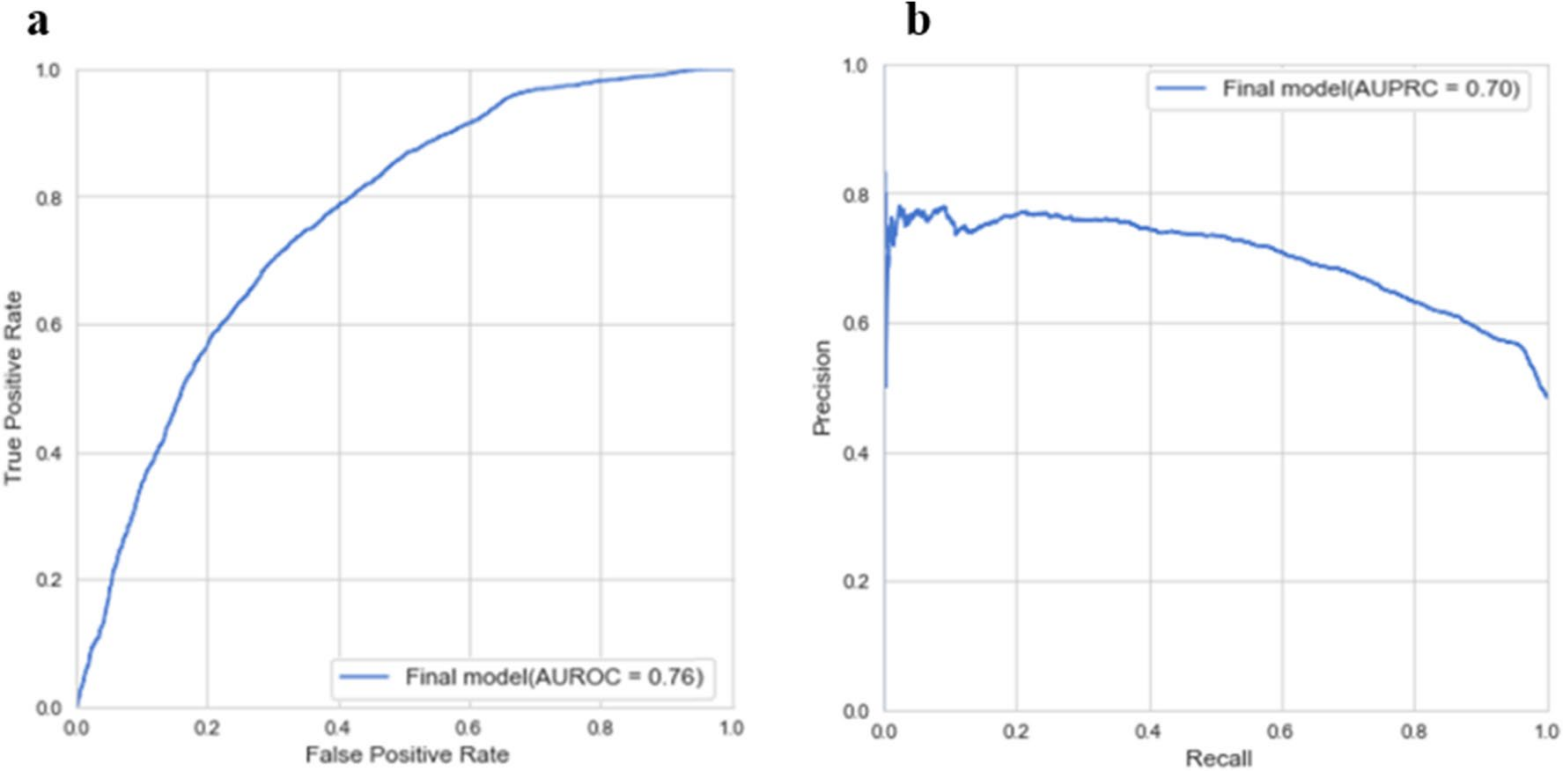
External validation of the final model on the TASMC data. (**a**) AUROC. (**b**) AUPRC.

The dataset from TASMC was used for external validation of the final model. The results (Fig. 4) show good performance with AUC 0.76 and AUPR 0.7, albeit less than in the development dataset. A certain reduction in performance is expected when validating a predictor on an independent data source. The slight decrease in performance here can be explained, in part, by the lower temporal resolution of the TASMC dataset, as well as by the higher rate of missing values.

## Discussion

We utilized machine learning models for predicting a deterioration event in the next 7–30 h based on EHR data of adult COVID-19 inpatients. Deterioration was defined as a high COVID-19 early warning score (mNEWS2 $\ge$ 7). On held-out data, the model achieved AUROC of 0.84 and AUPR of 0.74. The model was tested on an independent patient cohort from a different hospital and demonstrated comparable performance, with only a modest decrease. Using our predictor, we could anticipate deterioration of patients 7–30 h in advance. Such early warning can enable timely intervention, which was shown to be beneficial^5^.

Several previous studies have assessed the utility of machine learning for predicting deterioration in COVID-19 patients^14–18^; see also^19^ for a review. Most studies used strict criteria as their primary outcomes, such as mechanical ventilation, ICU admission, and death. However, the mNEWS2 score provides a more dynamic measure for clinical deterioration, allowing to trace patient conditions throughout the hospitalization. Since the mNEWS2 score is broadly adopted as a yardstick of COVID-19 inpatient status in medical centers around the world, we believe that demonstrating early prediction of high scores could provide valuable insights to physicians and bring to their attention particular patients that are predicted to be at high risk to deteriorate in the near future. Notably, our model can be readily adapted to other criteria for deterioration, e.g., mechanical ventilation or other mNEWS2 cutoffs.

Consistently with previous studies^14–18,20^, we confirmed the importance of known medical and inflammatory markers for severe COVID-19, such as age, body temperature, oxygen saturation, LDH and albumin. While most previous studies used only raw variables as features, our work emphasizes the importance of including summary statistics, such as the standard deviation of body temperature, for predicting the risk of COVID-19 deterioration. We note that, despite its previously reported importance^17,18,20,21^, C-reactive protein was excluded from our analysis since it was not consistently available in our data.

Most previous works that predicted deterioration utilized only baseline data, obtained on admission or a few hours thereafter^14–18^. Thus, they sought to predict the risk of a single deterioration event, possibly several days before its occurrence. Razavian et al. used data from the entire hospitalization period, but for prediction of favorable outcomes^22^. The novelty of our methodology lies in the fact that our model generates repeatedly updated predictions for each patient during the hospitalization, using both baseline and longitudinal data. This enables the identification of patients at risk throughout the hospitalization, while accounting for the temporal dynamics of the disease, allowing adjusted patient therapy and management. All predictions refer to events at least seven hours in advance, enabling early detection of patients at risk. Moreover, unlike many other prediction models, see^19^, our method was validated on data from a different center.

The final model used in this work was CatBoost, an algorithm for gradient boosting on decision trees. Such models have been successfully applied to various clinical applications^23–26^. They are often best performers for relatively small datasets, and have the additional advantage of being easily interpretable, an important factor in using machine learning models in the clinical setting^27^. Deep learning approaches often do better when powered by massive amounts of data^28–30^. With a larger sample size, we intend to take advantage of deep architectures in future work, including variants of recurrent neural network (RNN).

Our study has several limitations. First, it is retrospective, and model development was done based on data from a single center, which may limit its generalizability to external cohorts, especially considering the high variability of COVID-19 outcomes. Second, the mNEWS2 scores present a noisy signal, with frequent changes in the severity condition during the hospitalization. This impairs the score’s ability to be used as a robust predictor, compared to other approaches for predicting deterioration^15,31^, which use other signals, such as initiation of mechanical ventilation or death.

A potential concern is that a deteriorating patient will tend to have more frequent mNEWS2 measurements. This may bias our model and impair its adaptability to a general population of patients. To mitigate bias due to measurement intensity, we chose to exclude features that capture measurement frequency, although including them can improve performance. In addition, the training data had a majority of negative observations (~ 69%), showing that mild and modest conditions are well represented in the data. Furthermore, by summarizing measurements per hour we mask the measurement intensity within the same hour. Future work could examine time discretization over longer time windows and utilization of balancing techniques.

To date, only a few prognostic COVID-19 models have been prospectively validated or implemented in clinical practice^22,32^. The adoption of a model into clinical workflows requires the completion of several steps. First, to avoid site-specific learning, the model should be validated across several healthcare centers. Second, the model should be integrated into the institution’s EHR system, so that each variable is extracted from the database and fed into the pipeline in real-time. Third, prospective validation should be performed to assess the performance of the deployed model. Our study was done with future deployment in mind on several levels. It spanned two centers, with one used for validation only, and we plan to extend the study to additional centers. Collaborating with our clinical experts, we incorporated clinical standards into model development, for example when defining the inclusion and exclusion criteria and by addressing potential biases. In addition, by using SHAP values, we provided a decision support tool that could be interpretable to clinicians. Furthermore, the deterioration threshold (mNEWS2 cutoffs) and the prediction window (the time interval in the future for which the predictions are made), can be easily tuned, enabling tailored alarm policy for clinical setting (e.g., how often the alarm is raised). Future prospective validation is needed to assess the impact of the deployed model on patient outcomes.

In conclusion, machine learning-based prognostic tools have great potential for both care decisions and resource utilization in hospital wards. We described the development and validation of a model for the prediction of deterioration of COVID-19 inpatients within the next 7–30 h. In spite of the fact that the disease is novel and of high complexity, our model provides useful predictions for risk of deterioration, with good discrimination. Early detection and treatment of COVID-19 patients at high risk of deterioration can lead to improved treatment and to a reduction in mortality. Further validation of this vision is needed.

## Methods

### Cohort description

The development dataset consisted of all patients admitted to Sheba between March and December 2020 that tested positive for SARS-CoV-2. The validation dataset consisted of all patients admitted to TASMC between March and September 2020 who tested positive for SARS-CoV-2. The study was reviewed and approved by the Sheba Medical Center Institutional Review Board (number 20–7064) and by the Tel Aviv Sourasky Medical Center Institutional Review Board (number 0491–17), and conformed to the principles outlined in the declaration of Helsinki. All methods were performed in accordance with the relevant guidelines and regulations. Patient data was anonymized. The IRBs approved the waiver of informed consent.

The data used was extracted from longitudinal EHRs and included both time-independent (static) and temporal (dynamic) features from the entire hospitalization period. The static features were age, sex, weight, BMI and background diseases. The background diseases included hypertension, diabetes, cardiovascular diseases, chronic obstructive pulmonary disease (COPD), chronic kidney disease (CKD), cancer, hepatitis B and human immunodeficiency virus (HIV). The dynamic features include measurement of vital signs (including oxygen saturation), complete blood count (CBC), basic metabolic panel (BMP), blood gases, coagulation panel and lipids panel, including kidney and liver function tests, and inflammatory markers (Supplementary Table 2). Features with more than $40\%$ missing values or with zero variance were excluded. The temporal data was discretized to hourly intervals and multiple values measured within the same hour were aggregated by mean. We use the term *observation* for the vector of hourly aggregated feature values of the patient. An observation was formed if at least one measurement was recorded in that hour.

While our goal was to predict individual positive observations, in order to provide early warning, a closely related question is the prevalence of continuously deteriorating patients. To answer this question, we defined continuously deteriorating patients as those who had a period of 12 consecutive hospitalization hours with at least two mNEWS measurements, the majority of which had scores ≥ 7. 25.2% and 21.1% of the patients in Sheba and TASMC, respectively, satisfied this criterion. Notably, the correlation between mortality and deterioration according to this criterion was ~ 0.5 in both datasets.

### Inclusion and exclusion criteria

#### Inclusion criteria

Adult patients (age $\ge$ 18) with at least one mNEWS2 score.

#### Exclusion criteria

Patients who were in a severe state upon their admission, defined as having mNEWS2 score $\ge$ 7 in the first 12 h after admission (n = 156 patients). Observations from the  6 h period prior to a deterioration event, as we wish to predict at least  6 h in advance (n = 28,069 observations), and observations from the 8 h after the deterioration event (n = 5,157 observations). These two exclusion criteria defined the blocked prediction period during which no predictions are made (Supplementary Fig. 1). Observations where no mNEWS2 score was available in the next 30 h, for which predictions could not be compared to the true outcome (n = 9,812 observations). Patients with no laboratory results for BMP, CBC and coagulation during their entire hospitalization, since our model is based mainly on laboratory features (n = 15 patients). Patients' observations with $\ge$ 60% of the feature values missing (n = 424 observations).

### Outcome definition

The mNEWS2 scores were routinely calculated and updated in the EHR systems, as part of clinical care (see calculation protocol in Supplementary Table 1). The mean time period between two consecutive mNEW2 records was ~ 2.7 h in the development set before applying the inclusion and exclusion criteria, and ~ 2.5 h afterward. Observations with a high mNEWS2 score ($\ge$ 7) recorded in the next 7–30 h were called positive, and the rest were called negative. Notably, observations where no mNEWS2 score was available in the next 30 h were excluded (see “Inclusion and Exclusion Criteria”).

### Outlier removal

To remove grossly incorrect measurements due to manual typos or technical issues, we manually defined with clinicians a range of possible values (including pathological values) per each feature (Supplementary Table 4), and removed values outside this range. In total, 43,507 values were excluded.

### Data imputation

Missing values were observed mainly in lab tests and vital signs. We used linear interpolation for imputing missing data. The remaining missing data (e.g., missing values in observations that occurred before the first measurement of a feature, or features that were not measured for a patient at all) were imputed using the multivariate Iterative Imputer algorithm, implemented in the scikit-learn library in Python^33^, which was inspired by *MICE* (Multivariate Imputation by Chained Equation)^34^. The Iterative Imputer uses regression to model each feature with missing values as a function of other features, in a round-robin fashion. In each round, each of the features is imputed in this way. The dataset obtained in the final round serves as the final imputed dataset.

### Feature engineering

We created summary statistics over time windows of varying sizes to capture the temporal behavior of the data. The summary statistics were generated for 21 dynamic features that were reported as risk factors for severe COVID-19 in previous studies^17,20,21,35,36^ (Supplementary Table 4). We defined two time windows covering the last 24 and 72 h. For each time window, the summary statistics extracted were the mean, difference between the current value and the mean, standard deviation, minimum and maximum values. In addition, we extracted the same summary statistics based on the entire hospitalization period so far, with the addition of the linear regression slope (the regression coefficient). To capture recent data patterns, the difference and trend of the last two observed values ($({v}_{2}-{v}_{1}) {\mathrm{and}} \frac{{v}_{2}-{v}_{1}}{{t}_{2}-{t}_{1}}$ for values ${v}_{1},{v}_{2}$ recorded in times ${t}_{1},{t}_{2}$ respectively) were generated as well. In addition, to capture interactions between pairs of variables, we generated features for the ratios of each pair of variables in the risk factors subset (for example, neutrophils to lymphocytes ratio).

As imputation masks the information about the measurement frequency, we added features that capture the time since the last non-imputed measurement. While these features indeed improved our performance, the intensity of monitoring of a patient may reflect her medical condition (a deteriorating patient will tend to have more frequent measurements). As we aimed to predict deterioration when is not yet anticipated, we chose not to include these features in the developed model, since they can create bias due to measurement intensity.

We also added to the model unsupervised features that aimed to estimate how much an observation is irregular. We applied three anomaly detection approaches, One-Class SVM^37^, Isolation Forest^38^, and local outlier factor (LOF)^39^ to each hourly observation. Eventually, none of the anomaly features was included in the final model after the feature selection.

### Model development and feature selection

We performed a binary classification task for every hourly observation to predict deterioration in the next 7–30 h. Deterioration was defined as mNEWS2 $\ge$ 7. As deterioration can usually be predicted by a physician several hours in advance, based on signs and symptoms, observations from the six hours prior to the deterioration event were excluded (Supplementary Fig. 1). Once deterioration has occurred, no predictions were made in the next 8 h, and observations during that period were excluded. The length of the prediction window (30 h) and the blocked prediction windows (six hours before and eight hours after the event) were predefined with our clinical experts. These lengths can be easily tuned to fit other clinical settings. The predictions start with data collection (namely, on hospital admission), as long as the available data so far meet the inclusion and exclusion criteria, in terms of missing rate, blocked prediction windows and additional considerations (see “Inclusion and Exclusion Criteria”).

We evaluated ten supervised machine learning models for this prediction task: linear regression^40,41^, logistic regression, naïve Bayes, support vector machine (SVM)^42^, random forest^43^ and several algorithms for gradient boosting of decision trees, including XGBoost^44^ and CatBoost^45^. The hyperparameters of the models were determined using grid search over predefined ranges of possible values. The hyperparameter settings are listed in Supplementary Table 5. Data standardization was performed prior to model training when needed (for example, for SVM).

To handle the high dimensionality of our data after the feature engineering process, we examined two strategies or feature selection. The first selected the 100 features with the highest correlation with the target. The second used feature importance as calculated by XGBoost. Specifically, we trained XGBoost on the full imputed training dataset and used the computed feature importance scores to select the top 100 features for models training (Supplementary Table 6). Cross-validation of all algorithms was performed with the selected features, according to each strategy.

### Evaluation approach

We partitioned the development dataset into 80% training and 20% testing subsets (Supplementary Fig. 3). To avoid bias resulting from changes in clinical practice over time, the partition was done randomly across the hospitalization dates.

To estimate the robustness of the models on different patients and time periods, we used 20-fold cross-validation over the training set, and measured model performance using the area under the receiver-operator characteristics curve (AUROC) and the area under the precision-recall curve (AUPR). The testing set was used to evaluate the final model performance within the same cohort.

Finally, we used the validation dataset (TASMC) for external evaluation. The TASMC data had less frequent measurements than Sheba's. The slightly lower performance of the model on the TASMC cohort can be explained by its lower density and by the hourly discretization, which was chosen based on the Sheba data.

## Supplementary Information

Supplementary Information.

## Data Availability

Access to the data used for this study from Sheba and TASMC is restricted according to the Israeli Ministry of Health directives. Requests for access should be directed to Sheba and to TASMC.

## Code availability

The code used for data processing and model development is available at www.github.com/Shamir-Lab/covid19-mnews.
