# Supplementary material for: A machine learning model for predicting deterioration of COVID-19 inpatients
Source: Sci Rep. 2022 Feb 16;12:2630. doi: 10.1038/s41598-022-05822-7 (PMC8850417; doi:10.1038/s41598-022-05822-7)
Supplement: Supplementary file 1 — Supplementary Information. [file 41598_2022_5822_MOESM1_ESM.pdf]

Supplementary information

A Machine Learning Model for Predicting Deterioration of COVID-19 inpatients

Omer Noy[*,1], Dan Coster[*,1,2], Maya Metzger[1], Itai Atar[2], Shani Shenhar-Tsarfaty[2,3], Shlomo Berliner[2,3], Galia Rahav[2,4], Ori Rogowski[2,3], Ron Shamir[1]

[1] Blavatnik School of Computer Science, Tel-Aviv University, Tel-Aviv, Israel

[2] Sackler Faculty of Medicine, Tel-Aviv University, Tel-Aviv, Israel

[3] Department of Internal Medicine "C", Tel-Aviv Sourasky Medical Center

[4] Infectious Diseases Unit, Sheba Medical Center, Ramat Gan, Israel

* Equal contribution.

Corresponding Author: Ron Shamir

Email: rshamir@tau.ac.il

Phone Number: +972-54-304-3337

Postal address: School of Computer Science, Tel-Aviv University, 30 Haim Levanon Street, Tel-Aviv, Israel, 69978

Keywords: COVID-19, risk prediction, early detection of deterioration, machine learning, electronic medical records

SUPPLEMENTARY FIGURES AND TABLES

Supplementary Figure 1: Patient timeline from symptoms to deterioration. Data from the entire hospitalization period was utilized for model prediction, starting from the hospital admission. The green interval is when the deterioration predictions are made. Red areas represent blocked prediction periods during which no predictions are made: the six hours period prior to the deterioration event, and the eight hours period afterward. The length of the prediction window and the blocked prediction periods were predefined with clinical experts and can be easily tailored to fit other clinical settings.

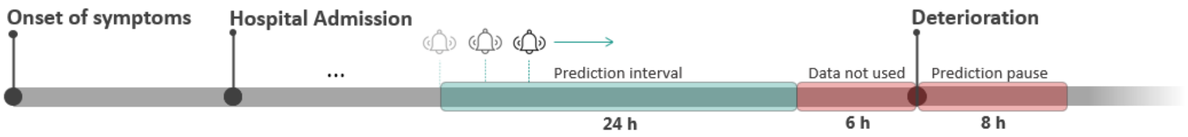

Supplementary Figure 2: Death and ICU admission rates as a function of the maximal mNEWS2 score during hospitalization in the development dataset.

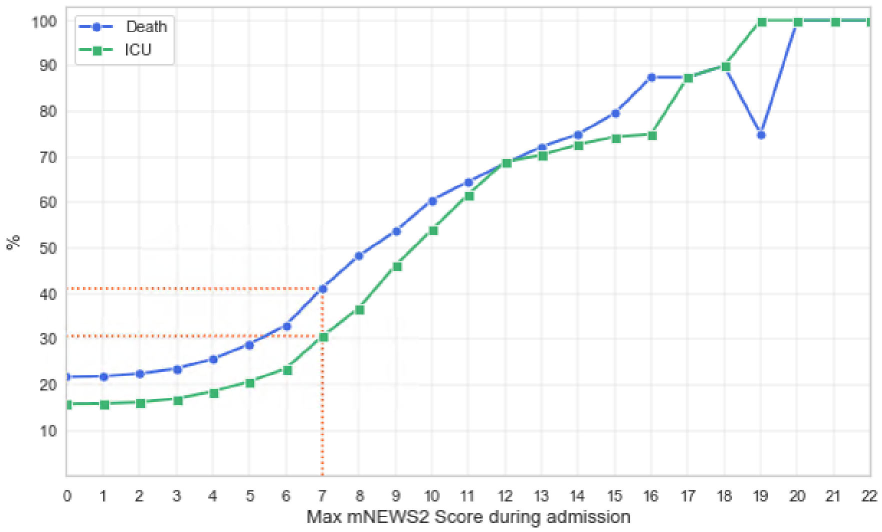

Supplementary Figure 3: Outline of the data partition and model development. First, the development dataset (Sheba) was split into 80% training and 20% testing subsets (1). To estimate the performance of 14 machine learning models, 20-fold cross-validation over the training set was performed (2). Then, the final model was trained over the entire training set (3) and evaluated on the testing set (4). External validation was done on the validation set (TASMC) (5).

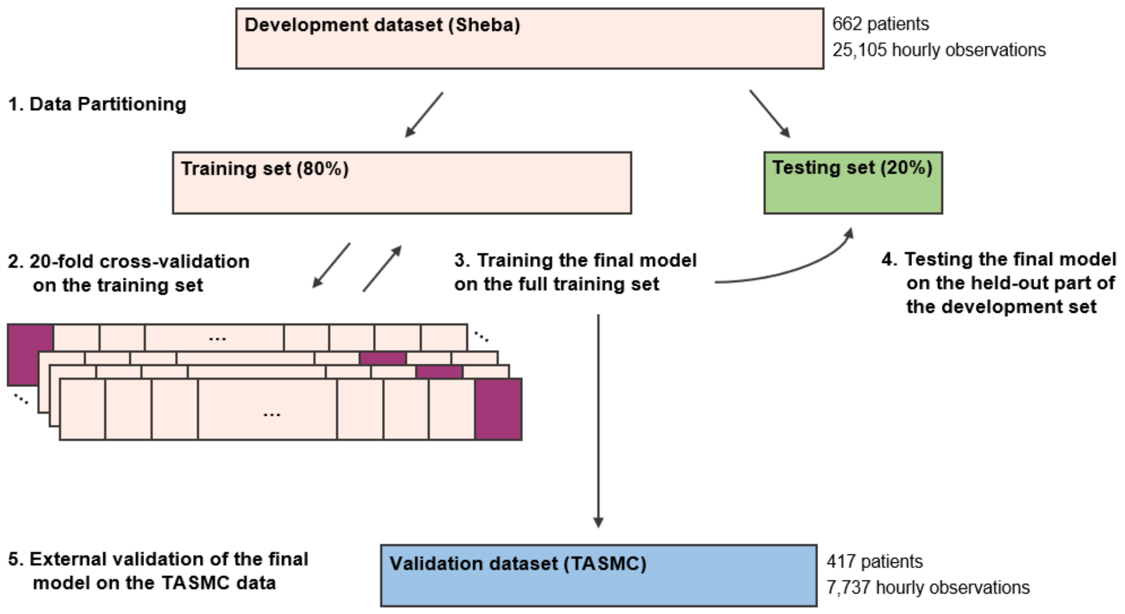

Supplementary Figure 4: Feature importance calculated by CatBoost ("PredictionValuesChange" metric). Features (rows) are ordered in decreasing overall importance. The importance score of a feature (x axis) is determined by how much on average the prediction changes when the feature value changes. A bigger change in the prediction value implies a higher importance.

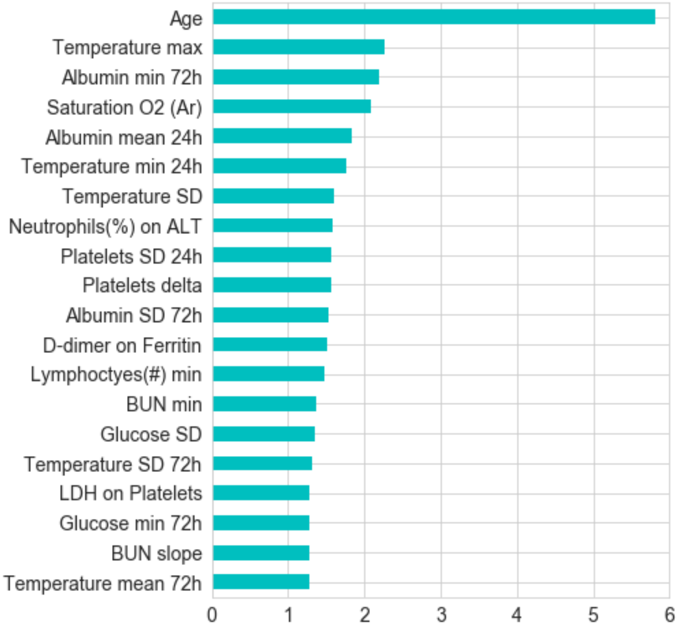

Supplementary Table 1: The mNEWS2 score. Scores are computed by summing the points for each category. This is an adapted version of the NEWS2 score, with the addition of 3 points for patients with age≥65 [10].

**Points**	**3**	**2**	**1**	**0**	**1**	**2**	**3**
Age				<65			≥65
Respiratory Rate	≤8		9-11	12-20		21-24	≥25
Oxygen Saturation	≤91	92-93	94-95	≥96			
Supplemental Oxygen		Yes		No			
Systolic BP	≤90	91-100	101-110	111-219			≥220
Heart Rate	≤40		41-50	51-90	91-110	111-130	≥131
Consciousness				Alert			Drowsiness<br>Lethargy<br>Coma<br>Confusion
Temperature	≤35.0		35.1-36.0	36.1-38.0	38.1-39.0	≥39.1	

**Score**	**Risk Grading**
0	-
1-4	Low
5-6 or 3 in one parameter	Medium
≥7	High

Supplementary Table 2: Table of Characteristics. Population characteristics for the two datasets used to develop and validate the model. Characteristics of both static features and first measurements of dynamic features are presented. P-values were calculated using Fisher's exact test and T-test for categorical and numerical values, respectively, and Bonferroni corrected for multiple comparisons. ’AR’ and ’V’ refer to arterial and venous blood, respectively.

**Variable**	**Development (Sheba Hospital)**		**Validation (TASMC)**		**P-Value**
	**N (%)**	**Mean ± SD**	**N (%)**	**Mean ± SD**	
Overall	**662**		**417**		
Age	662 (100.0%)	65.91 ± 16.86	417 (100.0%)	67.16 ± 18.01	1
Gender			1		
**Male**	391 (59.06%)		241 (57.79%)		
**Female**	271 (40.94%)		176 (42.21%)		
Morality	144 (21.75%)		73 (17.51%)		1
BMI	497 (75.08%)	28.21 ± 7.51	234 (56.12%)	27.37 ± 5.88	1
Weight	507 (76.59%)	81.11 ± 19.76	245 (58.75%)	77.25 ± 18.48	0.5385
Hypertension			<0.0001		
**Yes**	287 (43.35%)		60 (14.39%)		
**No**	375 (59.65%)		357 (85.61%)		
Diabetes			0.0046		
**Yes**	195 (29.46%)		83 (19.9%)		
**No**	467 (70.54%)		334 (80.1%)		
Cancer			0.0008		
**Yes**	91 (13.75%)		26 (6.24%)		
**No**	571 (86.25%)		391 (93.76%)		
Hepatitis B			1		
**Yes**	5 (0.76%)		3 (0.72%)		
**No**	657 (99.24%)		414 (99.28%)		
CKD			1		
**Yes**	43 (6.5%)		27 (6.47%)		
**No**	619 (93.5%)		390 (93.53%)		
HIV			1		
**Yes**	1 (0.15%)		1 (0.24%)		
**No**	661 (99.85%)		416 (99.76%)		
CVD			0.0046		
**Yes**	149 (22.51%)		58 (13.91%)		
**No**	513 (77.49%)		359 (86.09%)		
COPD			1		
**Yes**	26 (3.93%)		22 (5.28%)		
**No**	636 (96.07%)		395 (94.72%)		
HBA1C (#)	108 (16.31%)	45.34 ± 15.92	39 (9.35%)	54.11 ± 20.1	0.3497
HBA1C (%)	108 (16.31%)	6.3 ± 1.46	39 (9.35%)	7.1 ± 1.84	0.3629
Albumin	658 (99.4%)	35.81 ± 5.44	377 (90.41%)	38.35 ± 4.85	<0.0001
ALT	660 (99.7%)	33.02 ± 29.06	412 (98.8%)	37.47 ± 72.29	1
AST	661 (99.85%)	46.63 ± 43.84	378 (90.65%)	43.44 ± 35.46	1
BUN	662 (100.0%)	23.42 ± 16.66	417 (100.0%)	22.8 ± 19.15	1
Calcium	662 (100.0%)	8.82 ± 0.64	379 (90.89%)	8.67 ± 0.59	0.0098
CPK	644 (97.28%)	219.57 ± 595.46	380 (91.13%)	232.15 ± 451.02	1
Creatinine	662 (100.0%)	1.18 ± 0.98	417 (100.0%)	1.2 ± 1.36	1
Direct bilirubin	359 (54.23%)	0.28 ± 0.54	385 (92.33%)	0.26 ± 0.34	1
D-dimer	627 (94.71%)	3.22 ± 4.81	371 (88.97%)	2.19 ± 3.88	0.0244

Ferritin	471 (71.15%)	641.34 ± 1094.3	348 (83.45%)	799.82 ± 1280.3	1
Fibrinogen	577 (87.16%)	500.56 ± 174.18	320 (76.74%)	524.01 ± 145.45	1
Glucose	662 (100.0%)	135.34 ± 59.23	417 (100.0%)	128.16 ± 66.65	1
HCO3	597 (90.18%)	24.62 ± 3.78	341 (81.77%)	24.35 ± 4.33	1
HGB	662 (100.0%)	12.67 ± 2.14	417 (100.0%)	13.2 ± 1.94	0.0022
INR	656 (99.09%)	1.13 ± 0.21	416 (99.76%)	1.08 ± 0.21	0.0078
Lactate	600 (90.63%)	2.07 ± 1.07	178 (42.69%)	2.07 ± 1.33	1
LDH	656 (99.55%)	353.77 ± 215.99	367 (88.01%)	565.31 ± 269.78	<0.0001
Lymphocytes (#)	660 (99.7%)	1.18 ± 0.83	417 (100.0%)	1.18 ± 0.95	1
Lymphocytes (%)	662 (100.0%)	18.3 ± 11.77	417 (100.0%)	17.6 ± 12.02	1
Neutrophils (#)	662 (100.0%)	5.59 ± 3.86	417 (100.0%)	6.09 ± 4.27	1
Neutrophils (%)	662 (100.0%)	71.7 ± 14.19	417 (100.0%)	73.12 ± 13.49	1
NRBC	98 (14.8%)	1.24 ± 0.97	416 (99.76%)	0.2 ± 0.38	<0.0001
Osmolality (urine)	25 (3.78%)	368.0 ± 158.68	47 (11.27%)	451.17 ± 186.09	1
PO2 (AR)	56 (8.46%)	74.69 ± 27.7	341 (81.77%)	40.04 ± 36.73	<0.0001
PO2 (V)	596 (90.03%)	36.44 ± 27.54	81 (19.42%)	34.89 ± 35.42	1
PCO2 (AR)	56 (8.46%)	49.83 ± 11.69	342 (82.01%)	42.15 ± 9.08	<0.0001
PCO2 (V)	598 (90.33%)	43.12 ± 8.95	76 (18.23%)	42.67 ± 11.0	1
PH	541 (81.72%)	7.37 ± 0.08	341 (81.77%)	7.38 ± 0.07	1
Platelet	662 (100.0%)	208.46 ± 99.93	417 (100.0%)	202.05 ± 82.31	1
Potassium	661 (99.85%)	4.16 ± 0.61	417 (100.0%)	4.04 ± 0.59	0.0758
PTT	649 (98.04%)	30.88 ± 9.79	416 (99.76%)	32.32 ± 6.42	0.4093
RBC	662 (100.0%)	4.48 ± 0.75	417 (100.0%)	4.48 ± 0.69	1
RDW	662 (100.0%)	14.83 ± 2.23	417 (100.0%)	14.43 ± 1.68	0.0874
Sodium	662 (100.0%)	136.05 ± 5.69	417 (100.0%)	136.0 ± 5.74	1
Saturation O2 (AR)	592 (89.43%)	57.34 ± 24.21	247 (59.23%)	64.32 ± 26.61	0.0119
Total bilirubin	661 (99.85%)	0.67 ± 0.68	412 (98.8%)	0.6 ± 0.48	1
Triglycerides	367 (55.44%)	162.83 ± 118.33	309 (74.1%)	135.95 ± 67.14	0.0217
Troponin	575 (86.86%)	98.5 ± 950.41	412 (98.8%)	51.92 ± 319.07	1
VB12	312 (47.13%)	610.9 ± 421.27	292 (70.02%)	852.07 ± 511.04	<0.0001
WBC	662 (100.0%)	7.55 ± 4.48	417 (100.0%)	8.85 ± 14.28	1
Temperature	662 (100.0%)	37.016 ± 1.91	417 (100.0%)	37.63 ± 0.92	<0.0001
Pulse	662 (100.0%)	86.23 ± 16.66	417 (100.0%)	87.59 ± 17.23	1
Respiratory Rate	513 (77.49%)	19.84 ± 9.0	113 (27.1%)	20.98 ± 9.74	1
SBP	662 (100.0%)	131.7 ± 24.65	417 (100.0%)	136.94 ± 23.69	0.0295
DBP	662 (100.0%)	75.53 ± 13.23	417 (100.0%)	75.96 ± 15.34	1
Saturation	110 (16.62%)	94.7 ± 5.92	415 (99.52%)	92.75 ± 7.31	0.5151

Supplementary Table 3: Performance metrics of the final model on the testing set for different thresholds. PPV: positive predictive value, NPV: negative predictive value.

Threshold	Accuracy	Sensitivity	Specificity	PPV	NPV
**0.1**	0.66	0.88	0.56	0.48	0.91
**0.2**	0.74	0.78	0.71	0.56	0.87
**0.3**	0.77	0.69	0.80	0.62	0.84
**0.4**	0.79	0.60	0.87	0.69	0.82
**0.5**	0.79	0.55	0.91	0.73	0.81
**0.6**	0.79	0.48	0.94	0.79	0.80
**0.7**	0.80	0.44	0.97	0.87	0.79
**0.8**	0.78	0.34	0.98	0.90	0.76
**0.9**	0.73	0.17	0.99	0.93	0.72

Supplementary Table 4: Minimum and maximum accepted values of the dynamic features. Feature engineering was applied for the bolded features. ’AR’ and ’V’ refer to arterial and venous blood, respectively.

Feature	Min	Max	Units	Feature	Min	Max	Units
HBA1C (#)	0	240	mmol/mol	**Lymphocytes (%)**	0.2	100	%
HBA1C (%)	0	24	%	**Neutrophils (#)**	0.1	60	10e3/µL
**Albumin**	0	100	g/L	**Neutrophils (%)**	0.2	100	%
**ALT**	0	20000	U/L	NRBC	0	100	%
**AST**	0	20000	U/L	Osmolality (urine)	50	2000	mosmo/kg
Indirect bilirubin	0	20	mg/dL	**PO2 (AR)**	0	1000	mmHg
**Direct bilirubin**	0	20	mg/dL	PO2 (V)	0	1000	mmHg
BNP	0	10000	PG/ML	PCO2 (AR)	0	150	mmHg
Respiratory rate	1	100	BPM	PCO2 (V)	0	150	mmHg
**BUN**	2	200	mg/dL	PH	6.6	7.8	
Calcium	0	20	mg/dL	**Platelet**	0	1000	10e3/µL
CKMB	0	10000	U/L	Potassium	1	10	mmol/L
CPK	0	10000	U/L	**PTT**	5	200	Sec
CRP	0	1000	mg/L	Pulse	10	300	BPM
Creatinine	0	20	mg/dL	RBC	1	8	10e6/µL
DBP	20	240	mmHG	RDW	5	40	%
**D-dimer**	0	50	FEU mg/L	SBP	40	250	mmHG
**Ferritin**	0	20000	ng/ml	Sodium	110	200	mmol/L
**Fibrinogen**	0	1500	mg/dL	Saturation O2 (AR)	5	100	%
**Glucose**	0	2000	mg/dL	**Saturation**	5	100	%
HCO3	0	100	mmol/L	Total bilirubin	0	20	mg/dL
HGB	2	25	g/dL	**Temperature**	20	43	C°
INR	0.5	5		Triglycerides	10	2000	mg/dL
Lactate	0.2	15	mmol/L	**Troponin**	1	40000	ng/L
**LDH**	0	50000	U/L	Vitamin B12	100	2500	pg/ml
**Lymphocytes (#)**	0	20	10e3/µL	**WBC**	0.2	100	10e3/µL

Supplementary Table 5: Hyperparameters used in the models. Hyperparameter search grid and fixed hyperparameters used for the predictive models. The hyperparameter combinations were evaluated on each fold in the cross-validation and the average performance was computed. The optimal values used for the final model (CatBoost) appear in bold type. ‘poly’: polynomial kernel function; ‘rbf’: Radial basis function.

Model	Hyperparameter	Grid / Fixed value
**CatBoost**	Maximum number of trees	1,000
	Maximum depth	[6, **8**, 10]
	Learning rate	[0.001, 0.01, **0.03**, 0.1, 0.3]
	L2 Regularization coefficient	[1, 3, **5**]
**XGBoost**	Number of trees	100
	Maximum depth	[6, 8, 10]
	Learning rate	[0.001, 0.01, 0.03, 0.1, 0.3]
	colsample_bytree	1
**GBT**	Number of trees	100
	Maximum depth	[6, 8, 10]
	Learning rate	[0.001, 0.01, 0.03, 0.1, 0.3]
**Random Forest**	Number of trees	100
	Maximum depth	[6, 8, 10]
**Logistic Regression**	Regularization	[L1, L2]
**Linear Regression**	Regularization	[L1, L2]
**SVM**	Kernel	[‘linear’, ‘poly’, ‘rbf’, ‘sigmoid’]
**NB**	N/A	N/A

Supplementary Table 6: Top 100 features in importance as calculated by XGBoost. SD: standard deviation; /: ratio between two features. 24h,72h: time windows in which the statistic was computed. If the time window is not mentioned, the statistics is calculated on the entire hospitalization period up to the prediction time.

Age	Fibrinogen delta mean	Neutrophils (#) min
BMI	Fibrinogen delta mean 24h	Neutrophils (#) min 72h
Lactate	Fibrinogen max 72h	Neutrophils (#) / Glucose
Neutrophils (%)	Fibrinogen mean	Neutrophils (#) / Platelet
Sodium	Glucose mean	Neutrophils (#) trend
Saturation O2 - arterial blood	Glucose min 72h	Neutrophils (%) max
Albumin mean 24h	Glucose / Troponin	Neutrophils (%) max 24h
Albumin min 24h	Glucose SD	Neutrophils (%) max 72h
Albumin min 72h	LDH max	Neutrophils (%) min
Albumin / PTT	LDH max 72h	Neutrophils (%) min 72h
Albumin SD	LDH mean	Neutrophils (%) / ALT
Albumin SD 72h	LDH mean 72h	Neutrophils (%) / AST
ALT / Fibrinogen	LDH min 72h	Neutrophils (%) / D-dimer
AST min 72h	LDH / Albumin	Platelet delta
AST / Platelet	LDH / ALT	Platelet SD 24h
AST SD 72h	LDH / Platelet	Platelet SD 72h
BUN lr slope	LDH SD 72h	PTT lr slope
BUN delta mean 72h	Lymphocytes (#) min	PTT max 24h
BUN min	Lymphocytes (#) / D-dimer	PTT mean 72h
BUN / ALT	Lymphocytes (#) / Ferritin	PTT min 24h
BUN / Ferritin	Lymphocytes (#) / PTT	Temperature max
BUN / Troponin	Lymphocytes (#) SD 72h	Temperature mean 72h
BUN SD	Lymphocytes (%) max	Temperature min
D-dimer max	Lymphocytes (%) max 24h	Temperature min 24h
D-dimer max 72h	Lymphocytes (%) mean 72h	Temperature min 72h
D-dimer min	Lymphocytes (%) / AST	Temperature SD
D-dimer min 72h	Lymphocytes (%) / D-dimer	Temperature SD 72h
D-dimer / Albumin	Lymphocytes (%) / Fibrinogen	Troponin delta mean
D-dimer / AST	Lymphocytes (%) / PTT	Troponin SD 72h
D-dimer / Ferritin	Lymphocytes (%) SD	WBC min 24h
D-dimer / Fibrinogen	Lymphocytes (%) SD 24h	WBC / D-dimer
D-dimer / Platelet	Neutrophils (#) max	WBC SD
D-dimer SD	Neutrophils (#) max 72h	
Ferritin / Troponin	Neutrophils (#) mean	
